# 3D Printing in custom tray design for complete dentures in patients with flabby tissues

**DOI:** 10.3389/fdmed.2026.1603248

**Published:** 2026-05-08

**Authors:** Edgar Garcia Zea, Pablo Lenin Benitez Sellan

**Affiliations:** 1 Department of Prosthodontics, College of Dentistry and Dental Clinics, The University of Iowa, Iowa City, IA, United States; 2 School of Dentistry, Universidad de Especialidades Espiritu Santo, Samborondón, Ecuador

**Keywords:** denture, intraoral scanner, 3D printing, edentulous, removable prosthesis, flabby ridge, custom tray

## Abstract

**Background:**

Flabby ridge tissues present a significant challenge in complete denture fabrication because their mobility compromises support, stability, and retention. Conventional techniques such as the open window, selective pressure, or mucostatic methods attempt to control tissue displacement but often rely heavily on operator skill and may result in unpredictable outcomes.

**Case presentation:**

This case report describes a novel digital workflow for managing flabby ridge tissues during complete denture fabrication. An intraoral scan and design software were used to identify and duplicate the flabby ridge area. A 1.5 mm offset was digitally applied to provide controlled relief in the custom tray. The tray was manufactured through 3D printing, and clinical procedures included closed-mouth border molding followed by a functional definitive impression. This approach allowed the impression to be taken under occlusal load, reproducing the functional adaptation of the tissues.

**Results:**

The integration of digitally controlled relief with a closed-mouth mucocompressive technique provided accurate registration of the denture-bearing area while minimizing tissue distortion. The final prosthesis demonstrated satisfactory retention, stability, and esthetics. The patient reported comfort and functional efficiency, particularly during mastication.

**Conclusion:**

This case demonstrates the feasibility and advantages of combining digital design and 3D printing of custom trays with a closed-mouth impression technique in patients with flabby ridge tissues. The workflow offers a reproducible, minimally invasive, and time-efficient alternative to conventional approaches, highlighting the value of 3D printing as a tool to enhance precision and standardization in complete denture fabrication.

## Background

Complete dentures in patients with flabby tissues present unique challenges in prosthodontics. Flabby tissues, characterized by excessive mobility on the alveolar ridge, can compromise denture retention, stability, and support (1). Consequently, patients with flabby tissues require specialized impression techniques to ensure the stability and retention of their prosthesis. Conventional impression techniques may present challenges, as the requisite pressure for functional impressions can potentially displace movable tissues, thereby resulting in imprecise duplication (2).

To address this issue, a combined approach using both digital and conventional methods has been proposed. This combination allows for accurate recording of the flabby tissues while still capturing the functional aspects of the denture-bearing area (3). Among these techniques, the mucocompressive method proposed by Jiro Abe, which posteriorly displaces the flabby tissue during the impression process, has gained prominence due to its capacity to stabilize the tissue under masticatory forces and enhance overall denture retention (4). The integration of these techniques, along with digital technology, enables the fabrication of complete dentures that provide improved fit, stability, and patient comfort (5).

Several conventional approaches have been described for managing flabby ridge tissues, each with specific advantages and limitations. The open-window technique provides selective relief of mobile tissues but is highly technique-sensitive and may compromise the peripheral seal if not carefully performed (6). Surgical excision can permanently eliminate the flabby tissue, improving denture support, yet it is invasive, contraindicated in some patients, and may result in further ridge resorption (7). The selective pressure technique seeks to achieve balanced stress distribution by relieving compressible areas while recording firm tissues under load, though outcomes depend largely on operator skill and may lack reproducibility (8). Conversely, the mucostatic approach captures tissues at rest, minimizing distortion, but often leads to dentures with reduced stability and retention during function (9). These drawbacks emphasize the importance of exploring minimally invasive and reproducible alternatives, including digital workflows, to optimize outcomes in patients with flabby ridge tissues.

Recent advances in digital dentistry have enabled clinicians to combine conventional impression philosophies with modern CAD/CAM technologies (10). Intraoral scanners allow for detailed anatomical capture of the edentulous ridge, while 3D design software provides the ability to customize tray geometry with precise control over relief areas and material thickness (11). Furthermore, 3D printing has emerged as an efficient and accurate method for manufacturing custom trays, improving workflow reproducibility and reducing clinical chairside time (12). These innovations contribute to more consistent outcomes, especially in complex cases involving flabby ridge anatomy.

This article presents the design of a custom tray incorporating a digital workflow to optimize the mucocompressive closed-mouth impression and treatment of patients with flabby tissue. This innovative approach combines the benefits of digital technology with a proven mucocompressive technique, potentially enhancing the quality and efficiency of denture fabrication in patients with flabby tissues.

## Case presentation

A 65-year-old edentulous male patient, classified as ASA I and exhibiting a House philosophical attitude, presented to the clinic with complaints of inadequate denture retention and compromised function. He had been wearing the same set of complete dentures for over a decade.

**Figure 1:**
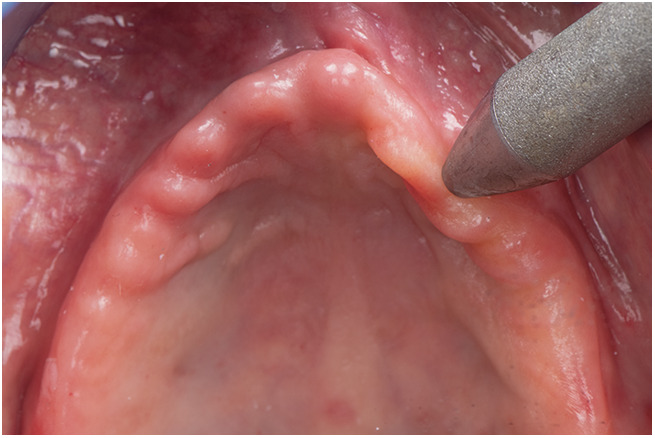
Maxillary arch showing flabby tissue region. This image depicts the anatomical site prior to scanning.

Intraoral examination revealed a flabby ridge in the anterior maxilla, without evidence of ulceration, inflammation, or infection. Anatomical inspection of the flabby region is shown in Figure 1. Various treatment options were considered, including tissue conditioning and surgical excision of the mobile tissue. However, after a thorough discussion of the risks and benefits, the patient expressed a clear preference for a conservative, non-surgical management strategy. The treatment plan involved the fabrication of a new maxillary complete denture using a custom impression tray designed through a digital workflow that incorporated the mucocompressive closed-mouth technique to accommodate the flabby tissue and enhance prosthetic fit.

**Figure 2:**
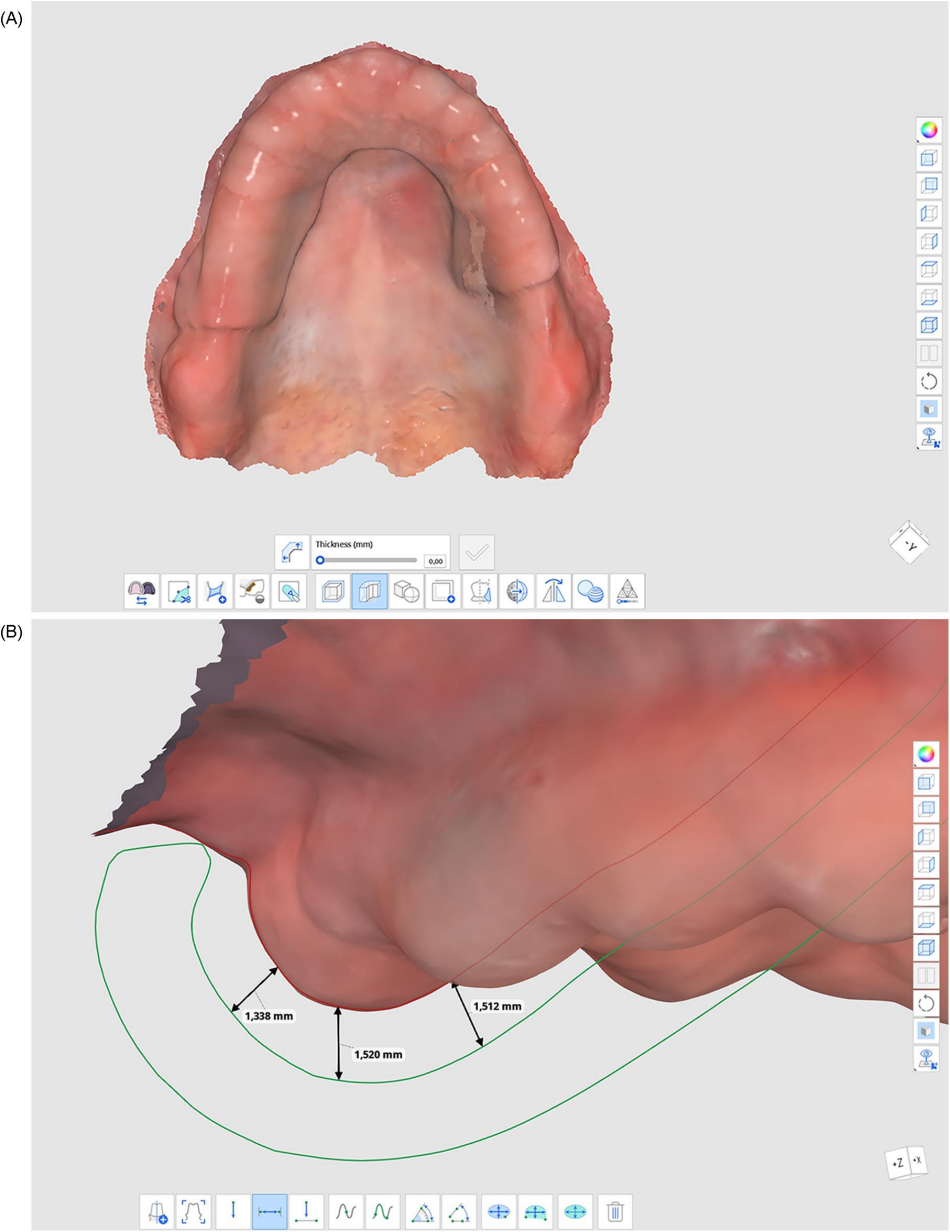
**(A)** thickened region on the 3D model representing the area of flabby tissue; **(B)** measurement parameters for the thickness increase applied to the flabby tissue area.

An intraoral scan of the edentulous maxillary arch was performed using a Medit i700 scanner (Medit Corp), ensuring that no pressure was applied during the scanning procedure to prevent distortion of the mobile tissue. The scan was initiated at the mid-palatal region and proceeded distally along the alveolar ridge. Buccal and palatal vestibules were captured last to minimize distortion of flabby tissue. The ’Smart Scan Filtering’ function in Medit was deactivated to ensure preservation of the flabby tissue contours without algorithmic correction. The digital file was evaluated using Medit Design's color map feature and visually inspected for completeness and absence of stitching errors. The scanned file was imported into Medit Design (Medit Corp), where the 3D model was inspected and aligned with anatomical landmarks. The flabby tissue region was outlined using the Duplicate tool, and a duplicate mesh of this region was generated (Figure 2A).

Within the duplicated mesh, the Thicken function was applied to expand the outlined area by 1.5 mm in both height and width (Figure 2B). Although a 1.5 mm thickening was targeted, variations from 1.3 to 1.52 mm occurred due to the natural curvature of the anatomical surface and the freeform sculpting tools within the software. The flabby tissue zone was manually delineated by the operator using the ‘Duplicate’ tool, allowing selective modification of that region for controlled tissue compression. This modification was made to compensate for the compressibility of the flabby tissue during functional impression-making, allowing for more predictable adaptation. The thickened mesh was then merged with the original maxillary model using the Boolean Union tool, creating a unified anatomical model that reflected the prosthetically significant tissue characteristics. The Sculpting tool was subsequently employed to refine the transitional zones between modified and unmodified regions, smoothing the contours to eliminate sharp edges that could interfere with tray adaptation.

**Figure 3:**
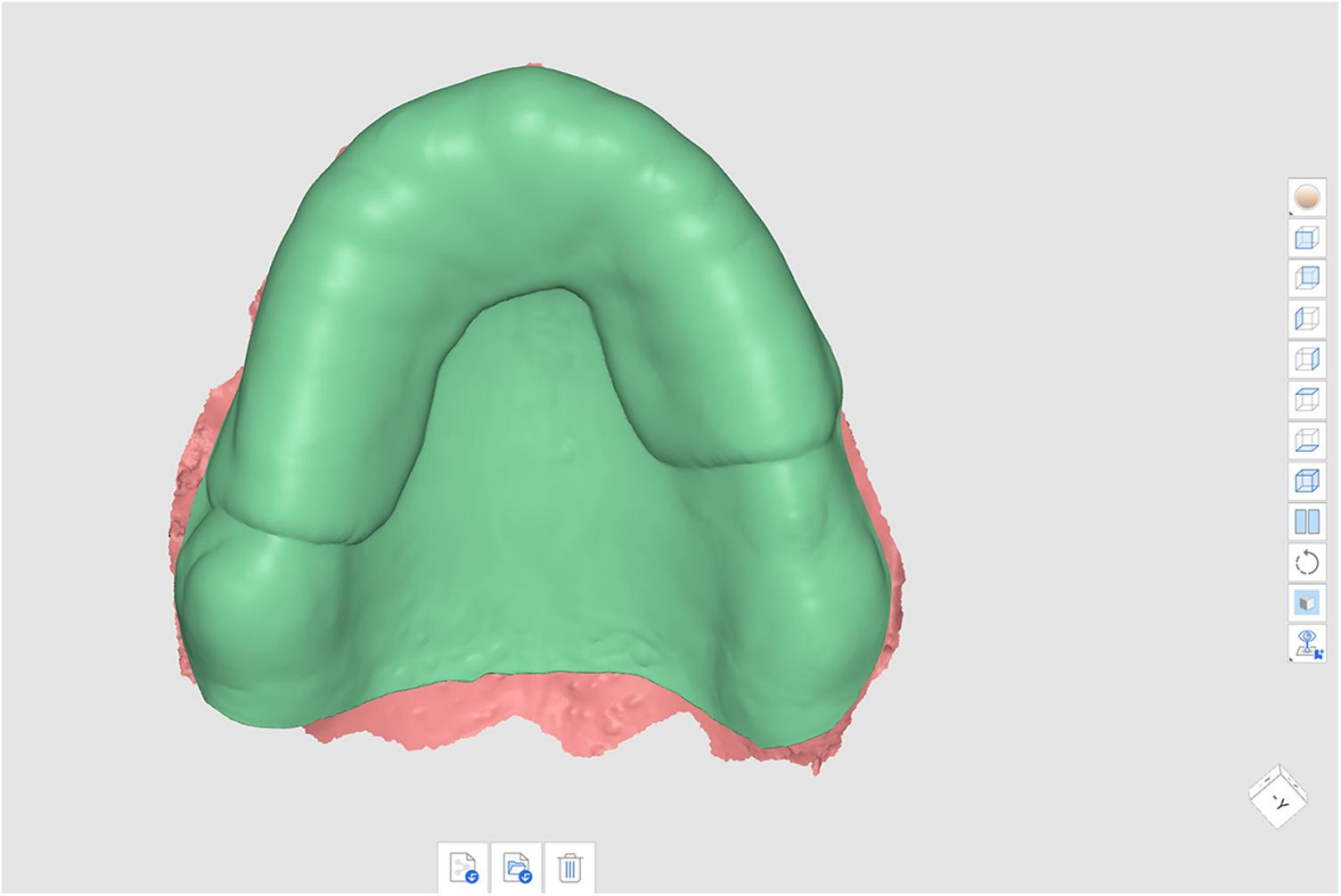
Custom tray design with defined tray boundaries and a 2 mm offset to control impression material application.

**Figure 4:**
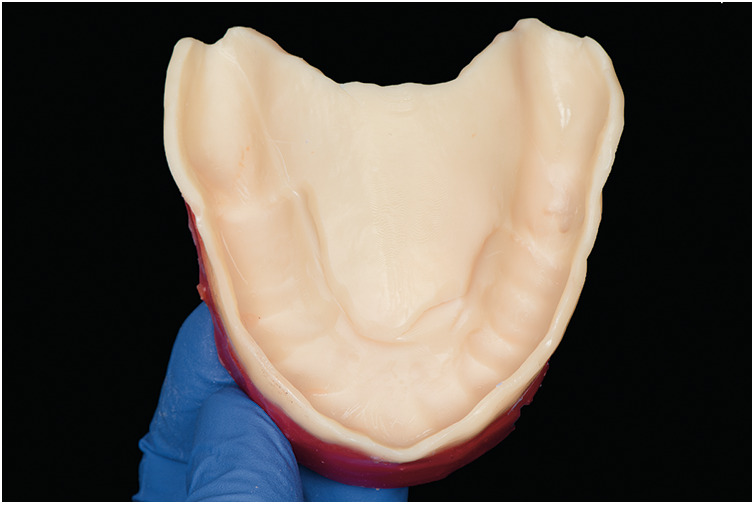
Custom tray fabricated using biocompatible 3D printed resin.

Next, the custom tray design boundaries were outlined using the Custom Tray Design feature within the software. The selected region was duplicated, and a 2 mm uniform offset was added using the Thicken function to provide adequate space for impression material (Figure 3). In Sculpting Mode, retention features such as grooves and dimples were incorporated using the Add Mesh function, enhancing mechanical interlocking of the impression material. Once finalized, the custom tray design was exported in STL format and 3D printed using a DLP printer (Shining 3D AccuFab-CEL; Shining 3D Tech, China). The print was performed using a biocompatible resin (Soflex Prov A2; W2P Engineering, Austria). The tray was oriented horizontally on the build platform to maximize accuracy of the intaglio surface and printed at 100 μm layer thickness (Figure 4).

**Figure 5:**
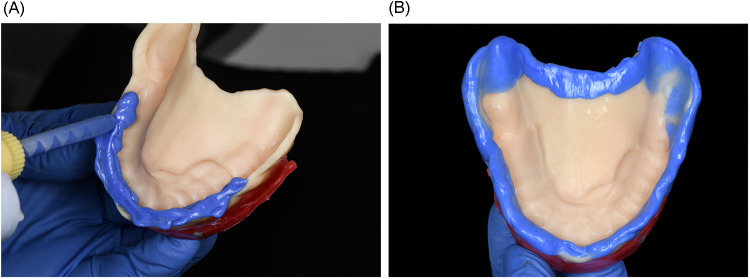
**(A)** border molding using heavy-body PVS in the anterior region of the tray; **(B)** completed border molding including the posterior flange area.

**Figure 6:**
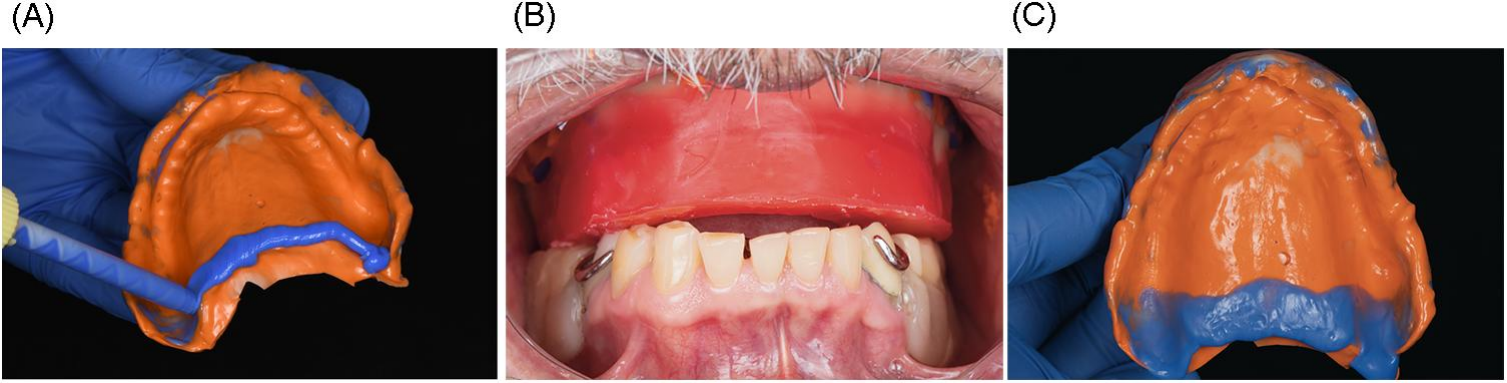
**(A)** additional posterior pressure applied during border molding to enhance tissue compression in closed-mouth impression; **(B)** intraoral view of the definitive closed-mouth impression with light-body PVS; **(C)** final wash impression showing precise capture of both functional and flabby tissues using the closed-mouth impression technique.

After printing, the tray was washed in 96% isopropyl alcohol using an ultrasonic bath for 5 min (2.5 min per cycle). It was then air-dried and inspected for any residual uncured resin. Post-curing was performed in the Shining 3D FabCure 2 unit, a high-performance UV curing system designed for dental applications. The tray was cured for a total of 10 min (5 min per side) using multi-LED 405 nm UV light, ensuring uniform polymerization and dimensional stability as per the manufacturer's instructions. For the preliminary impression, heavy-body polyvinyl siloxane (Heavy Body VPS; PlastCare USA) was applied to the anterior portion of the tray. The tray was inserted intraorally, maintaining stable posterior support to ensure correct seating while the material set. Once polymerized, any excess material extending over the ridge base was carefully trimmed to avoid displacing the flabby tissue during the final impression (Figure 5A). Border molding was completed first in the anterior region and then extended to the posterior, with additional pressure applied to the posterior flanges to enhance functional seal during closed-mouth impression-making (Figures 5B, 6A). Light-body VPS (PlastCare USA) was then applied evenly to the tray, and the patient was instructed to bite gently in the closed-mouth position, achieving optimal tissue compression and functional registration (Figure 6B).

**Figure 7:**
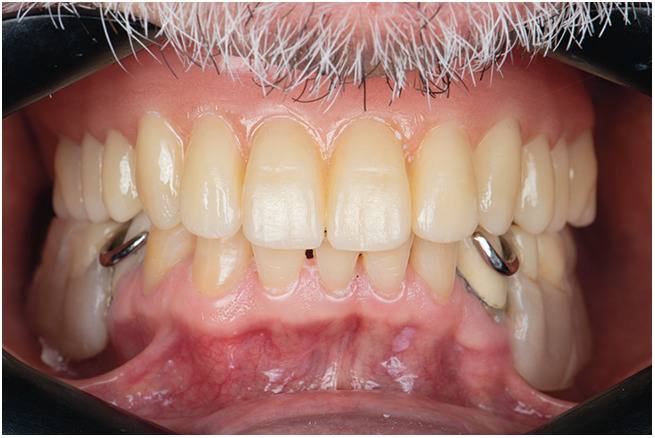
Final denture adaptation and delivery.

Following polymerization of the light-body material, the tray was removed, and the definitive impression was evaluated for detail, tissue support, and extension (Figure 6C). The final prosthesis was fabricated based on this master impression, and upon delivery, it demonstrated satisfactory retention, support, and patient comfort (Figure 7). After denture delivery, the patient was instructed to maintain hygiene using a soft denture brush and non-abrasive cleanser, and to remove the denture at night. Chlorhexidine rinses were recommended during the adaptation phase. Follow-up was scheduled at 24 h, 1 week, and at 3- to 6-month intervals to monitor soft tissue health, adaptation, and patient satisfaction.

## Discussion

The management of flabby ridges in complete denture fabrication requires precise impression techniques that balance tissue compression and anatomical accuracy (13). Conventional methods, including selective pressure and mucocompressive techniques, have achieved clinical success; however, their dependence on the clinician's manual dexterity can lead to variability in treatment outcomes (14). Numerous techniques have been developed with the shared objective of preserving tissue morphology by limiting distortion caused by tray pressure and impression material flow (15, 16). Among these, the use of a custom open-window tray combined with selective pressure techniques has gained broad clinical acceptance due to its ability to selectively relieve mobile areas while maintaining peripheral support (17).

Abe et al. introduced a mucocompressive philosophy that contrasts with techniques aimed at avoiding pressure on flabby tissues (4). Recognizing that hypertrophic tissues may be displaced anteriorly by occlusal forces, their method employs a custom tray with selective wax relief in the flabby region to strategically direct pressure during border molding (2). The protocol begins with anterior molding that intentionally displaces the mobile tissue posteriorly to enhance peripheral seal, followed by posterior molding and a definitive closed-mouth impression (4). This approach not only improves denture retention and stability but may also support long-term tissue adaptation. When combined with a balanced occlusion, the technique helps distribute masticatory loads more uniformly, reducing trauma, enhancing comfort, and potentially contributing to gradual reduction of flabby tissue volume through consistent mechanical support and diminished inflammation.

In contrast to conventional techniques such as the open-window tray or the use of wax relief, which depend heavily on intraoral adjustments and operator technique, the digital workflow allows for reproducible control of tray thickness and tissue relief through software design (16). Although no standardized clinical outcome measures were applied in this case, the patient reported high levels of comfort, retention, and functional stability during follow-up, supporting the clinical viability of the approach.

Recent reports illustrate different approaches to managing excessively movable tissues in complete denture patients. Conventional techniques, such as the Hobkirk method and its modifications, remain widely used to minimize tissue displacement with selective relief and window trays, but they are highly technique-sensitive and lack reproducibility (18–20). More recently, digital and hybrid workflows have been proposed. Park et al. combined intraoral scanning of the flabby anterior maxilla with a conventional closed-mouth impression, demonstrating feasibility but requiring complex data merging and reliance on proprietary CAD software (3).

Compared with these approaches, our workflow shares the use of intraoral scanning, CAD customization, and 3D printing, but it differs by incorporating a digitally controlled relief offset integrated with a functional closed-mouth impression, all designed through open-source software. This not only reduces dependency on manual adjustments or proprietary platforms but also enhances reproducibility and accessibility. In this way, our method adds value by addressing common limitations of both conventional and previously reported digital techniques, providing a standardized yet practical alternative for clinical use.

The integration of digital workflows in custom tray design provides a significant improvement in controlling the selective pressure applied during impression-making (21). One of the main advantages of this workflow is the integration of digitally controlled relief customization with a reproducible closed-mouth mucocompressive impression technique. This combination ensures consistent and controlled pressure on flabby tissues, minimizes distortion, and enhances both functional adaptation and reproducibility compared to conventional or purely manual methods (11). Moreover, 3D printing technology allows for the reproducibility of trays, minimizing variability between impressions and improving standardization in clinical practice (22).

Studies have shown that 3D-printed trays can achieve similar or superior accuracy to conventional ones when properly designed and post-processed (12). Their low cost and fast turnaround allow chairside adjustments to be minimized. However, limitations include the need for training in digital tools, access to 3D printers, and careful resin handling to avoid distortion due to post-curing shrinkage. Proper orientation during printing (e.g., horizontal positioning) and complete light curing are critical to avoid dimensional changes (23). In the context of denture tray fabrication, the build angle has been shown to significantly influence the final product's accuracy. Specifically, printing at 0° or 45° has consistently produced more accurate results, while 90° orientations tend to introduce greater distortion and require longer print times (24).

In addition to this, it reduces chairside time and minimizes the need for repeated adjustments. The ability to visualize and modify the tray design before fabrication ensures that errors are minimized, and the final impression captures the necessary anatomical landmarks accurately (12). Additionally, the reduced dependency on manual adaptation improves the overall precision of the impression process, which is particularly beneficial for clinicians treating complex edentulous cases (25).

Despite these advantages, the digital workflow does present challenges. The accuracy of intraoral scanning in edentulous patients remains a limitation due to difficulties in capturing soft tissue movement (26). Ensuring adequate tissue stabilization during scanning is critical to achieving high-quality digital impressions. Furthermore, accessibility to digital equipment and familiarity with CAD software may pose initial barriers for clinicians transitioning from conventional techniques (27).

Future advancements in artificial intelligence-driven CAD tools and intraoral scanning technologies could further refine the precision of digital workflows for denture fabrication (28). The 1.5 mm relief thickness used in the flabby ridge region was based on empirical clinical judgment. It was not derived from direct *in vivo* measurements of mucosal compressibility. Future studies using intraoral sensors or pressure mapping technologies are necessary to validate and refine this digital parameter. As this is a single case report, the generalizability of the findings is inherently limited. Further clinical studies are encouraged to evaluate the reproducibility and broader applicability of this digital workflow.

## Conclusion

The integration of digital workflows in custom tray design for patients with flabby tissues offers a meaningful improvement in workflow reproducibility and control over selective tissue relief in complete denture fabrication. By leveraging 3D scanning and CAD technologies, clinicians can achieve precise control over impression techniques, improving the accuracy and efficiency of complete denture fabrication. While some limitations remain, ongoing technological developments are likely to enhance the feasibility and reliability of this approach in routine prosthodontic practice.

## Data Availability

The original contributions presented in the study are included in the article/Supplementary Material, further inquiries can be directed to the corresponding author.
