# Supplementary figures and images for: 3D Printing in custom tray design for complete dentures in patients with flabby tissues
Source: Front Dent Med. 2026 May 8;7:1603248. doi: 10.3389/fdmed.2026.1603248 (PMC13194386; doi:10.3389/fdmed.2026.1603248)

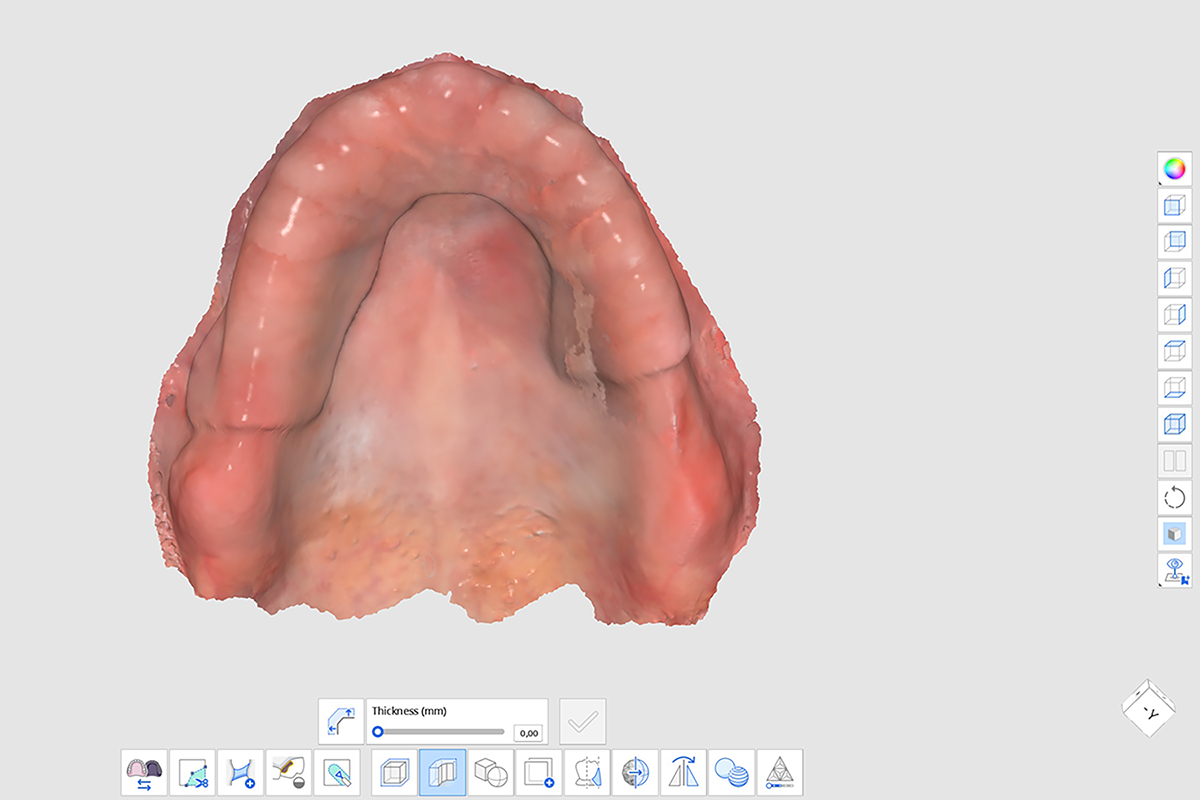

Supplement: Supplementary file 1 [file Datasheet1.zip › Figure 2A.tif]

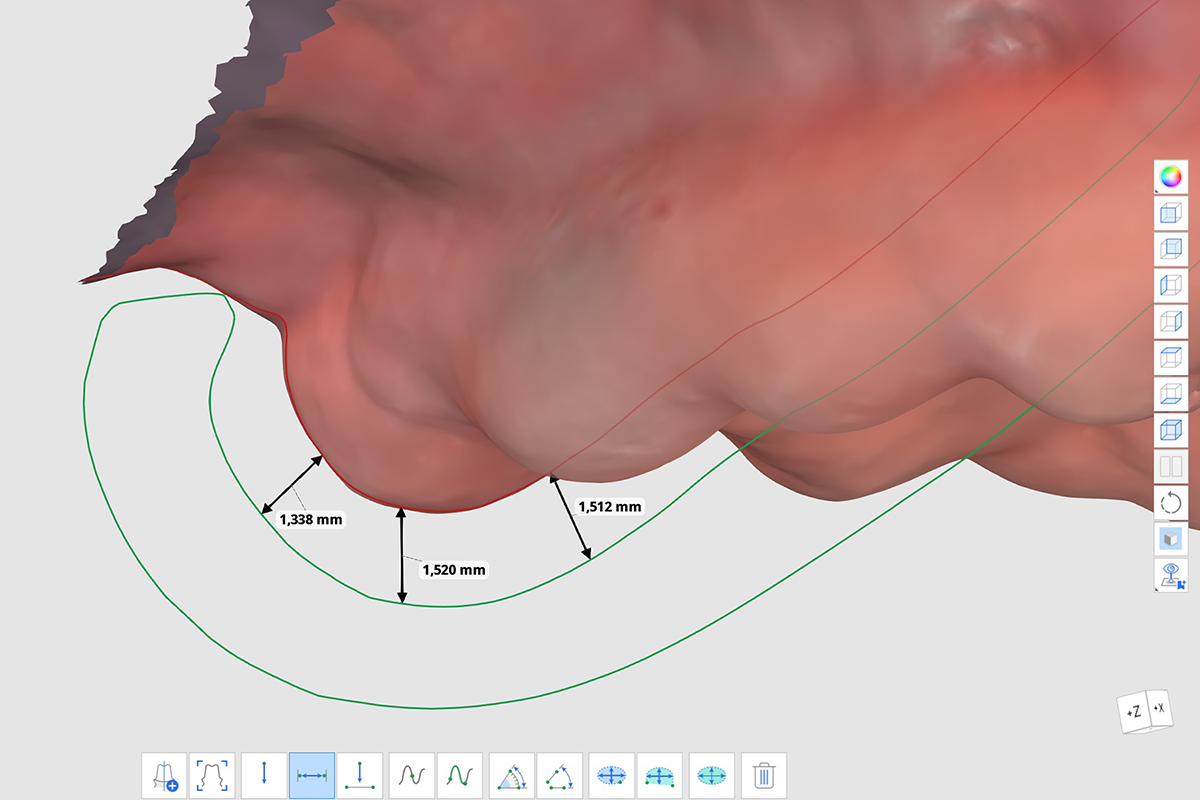

Supplement: Supplementary file 1 [file Datasheet1.zip › Figure 2B.tif]

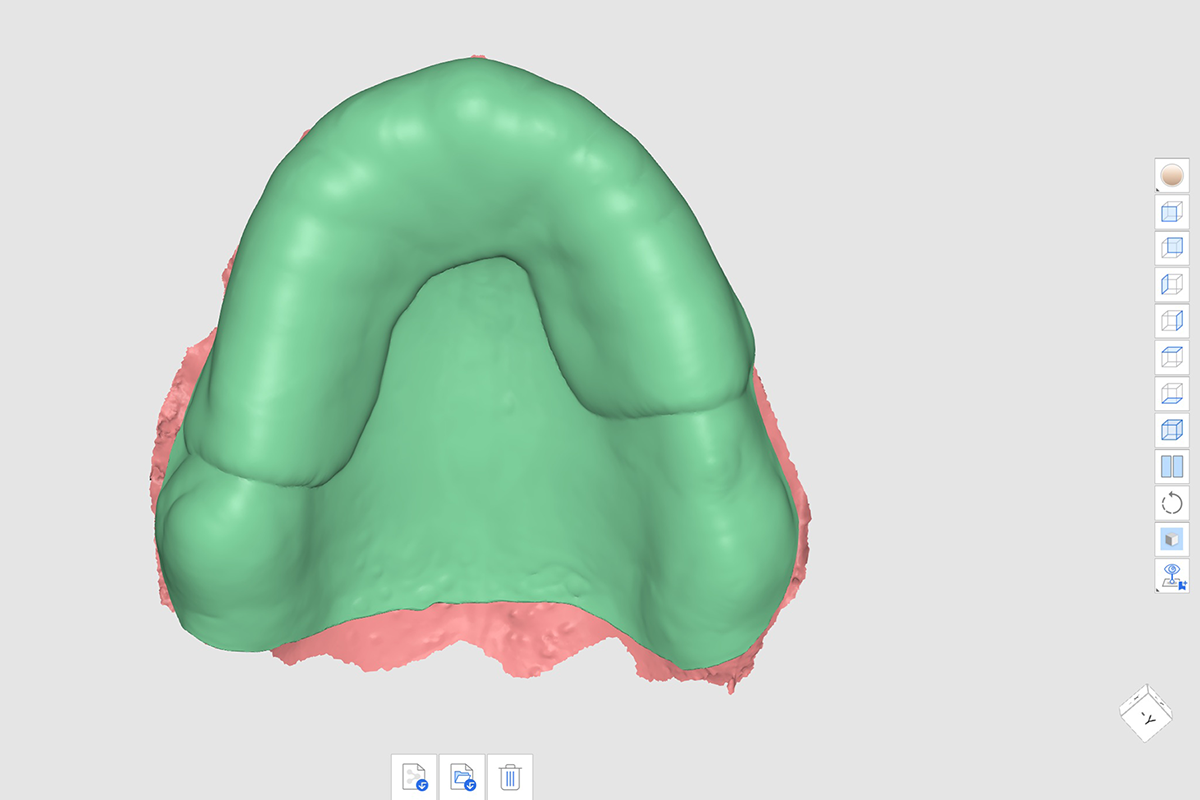

Supplement: Supplementary file 1 [file Datasheet1.zip › Figure 3.tif]
